# Supplementary material for: Colloidal CdSe nanocrystals are inherently defective
Source: Nat Commun. 2021 Feb 9;12:890. doi: 10.1038/s41467-021-21153-z (PMC7873310; doi:10.1038/s41467-021-21153-z)
Supplement: Supplementary file 1 — Supplementary Information [file 41467_2021_21153_MOESM1_ESM.pdf]

Supplementary Information for: Colloidal CdSe Nanocrystals are Inherently Defective

Tamar Goldzak[1,2,†], Alexandra R. McIsaac[1,†], and Troy Van Voorhis[1,*]

[1]Department of Chemistry, Massachusetts Institute of Technology, Cambridge, MA 02139
[2]Current address: Department of Chemistry, Columbia University, New York City, NY 10027
[†]These authors contributed equally to the work.
[*]E-mail: tvan@mit.edu

Supplementary Note 1

Structural Analysis To perform the structural analysis and identify undercoordinated atoms, we first plotted a histogram of Cd-Se and Cd-ligand distances. We used this histogram to identify a cutoff for which atoms should be considered "bonded," which varies slightly based on the system in question (3.0 $\mathring{A}$ for the $Cd_{33}Se_{33}$ and $Cd_{38}Se_{38}$ systems, and 3.3 $\mathring{A}$ for the $Cd_{91}Se_{91}$ system). Once an appropriate cutoff has been selected, we count the number of atoms that each Cd and Se atom is bonded to. For Se, we consider only Cd's to be "bonded," while Cd can be bonded to either an Se or the attaching atom of the ligand (e.g. N for $MeNH_2$). Any atom that is bonded to 2 or fewer other atoms is considered "undercoordinated."

After identifying any undercoordinated atoms, we used the excited state Löwdin charges to determine if these undercoordinated atoms played a role in creating surface traps. We sum the fraction of charge on the undercoordinated atoms for each excitation, and compare this charge to the amount of charge we would expect to be on each atom if the electron and hole were uniformly distributed over all of the atoms. We then plot the charge fraction of the electron and the hole on undercoordinated atoms, for each excitation.

In Supplementary Figure 1, we show the histogram of Cd-Se and Cd-ligand distances for the $Cd_{91}Se_{91}$ NC with methylamine ligands. For this system, we chose a cutoff of 3.3 $\mathring{A}$, , and we find that there are 8 undercoordinated Se atoms and no undercoordinated Cd atoms.

We plot the charge analysis of the hole/electron on the undercoordinated Se atoms in Fig. 4 of the main text and Supplementary Figure 2, respectively. For the hole charge analysis, we find that the low-energy excitations $E_{\mathrm{ex}} < 2.95$ eV have up to 86% of the charge from the excited hole on the undercoordinated Se atoms, with 45% of these states having $60 - 86\%$ of the hole charge located on the undercoordinated Se atoms. Even for higher

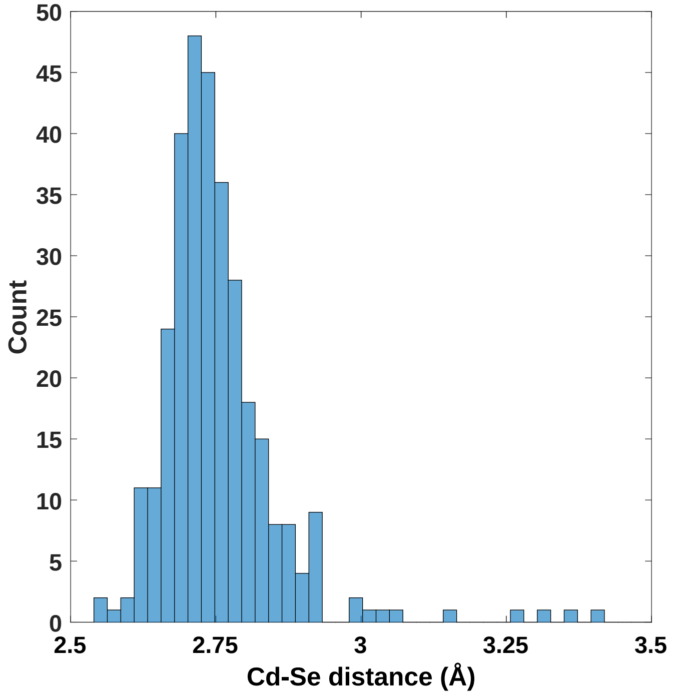

Supplementary Figure 1: Histogram of Cd-Se distances in the $Cd_{91}Se_{91}$ NC with $MeNH_2$ ligands.

energy excitations $E_{\mathrm{ex}} > 2.95$ eV, these undercoordinated atoms have a disproportionate amount of charge. 21% of these states have $60-86\%$ of the charge on the undercoordinated Se, and 15% of the states have $40-60\%$ of the charge on them. This is much higher charge than would be expected from a uniform distribution of charge.

For the electron charge analysis, we find that the low-energy excitations $E_{ex} < 2.95$ eV have up to 83% of the charge from the excited electron on the undercoordinated Se atoms. Unlike the hole charge, the excited electron has only 13% of states with $60-83\%$ of the electron charge located on the undercoordinated Se atoms. For higher energy excitations $E_{ex} > 2.95$ eV, these undercoordinated Se atoms have 3% of states with $60-83\%$ of charge on them. The percentage of states with high electron charge on the undercoordinated Se is generally low, but we wouldn't expect the electron to localize on undercoordinated Se atoms at all.

When decreasing the cutoff to 3.1 $\AA$ which roughly corresponds to the first peak of Cd-Se distances, there is 1 undercoordinated Cd atoms, and the number of Se atoms remains the same. There is no appreciable electron charge located on the undercoordinated Cd atom. For the hole charge there is some charge localized on this undercoordinated Cd atom but its magnitude is small and thus its effect is not pronounced.

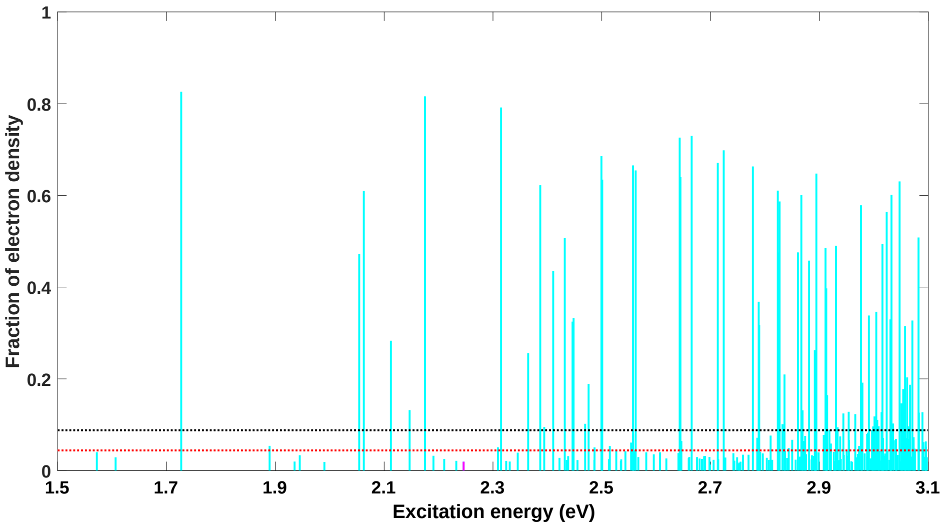

Supplementary Figure 2: Fraction of electron density on 2-coordinate Se atoms in $Cd_{91}Se_{91}$ with methylamine ligands. The magenta peak corresponds to excitation #16 (see main text Fig. 4). The red dashed line indicates the expected fraction on the 2-coordinate Se atoms if the density were evenly distributed across all Cd and Se atoms, and the black dashed line indicates the expected fraction on the 2-coordinate Se atoms if the density were evenly distributed across all Se atoms.

Supplementary Note 2

Band structure We calculated the orbital density of states (DOS) for $Cd_{33}Se_{33}$, $Cd_{38}Se_{38}$, and $Cd_{91}Se_{91}$ with methylamine ligands. The band structures were calculated from the orbital energies, applying Gaussian broadening of $\sigma = 0.12$ eV. The plots are presented in Supplementary Figure 3. One can see that as the NC size increases the HOMO-LUMO gap decreases, as expected from the particle in a sphere model. For the smallest NC ($Cd_{33}Se_{33}$) the HOMO and LUMO orbitals are separated from the more denser DOS of the occupied and virtual orbitals; as the size increases there are more orbitals close to the HOMO and LUMO orbitals, and the separation of the HOMO and LUMO from the denser part of the DOS is less clear.

We also calculated the DOS for the $Cd_{38}Se_{38}$ NC with Me_3PO ligands and no ligands, which are compared with $Cd_{38}Se_{38}$ with methylamine ligands in Supplementary Figure 4.

Supplementary Note 3

Basis set comparison In order to ensure that our results were not an artifact of the basis set used, we also conducted TDDFT calculations on the $Cd_{38}Se_{38}$ NC using a larger basis set on the ligands. In Supplementary Figure 5, we show the orbital density of states with the standard LANL2DZ basis set used in the main text, as well as with LANL2DZ on the Cd and Se and 6-31G* on the ligands. The two are nearly indistinguishable, with

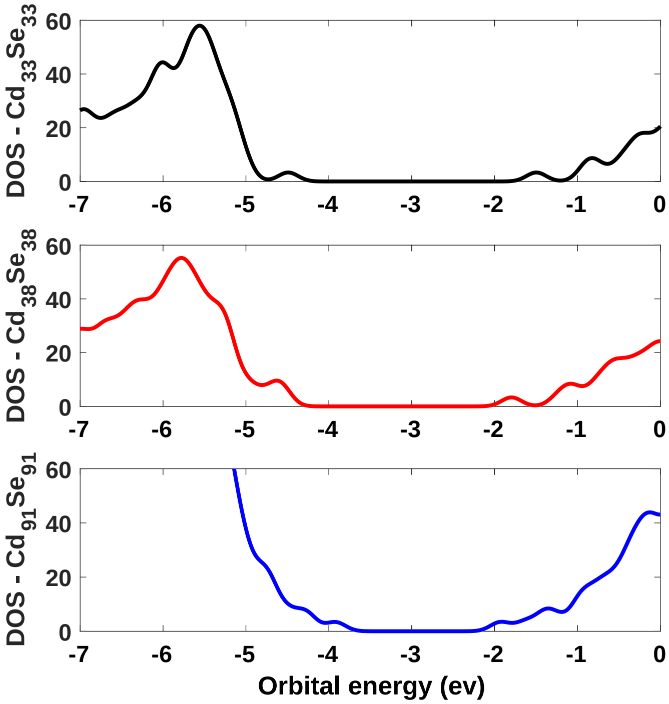

Supplementary Figure 3: Orbital density of states for $Cd_{33}Se_{33}$ (top), $Cd_{38}Se_{38}$ (middle), $Cd_{91}Se_{91}$ (bottom) with methylamine ligands, using the standard LANL2DZ basis set

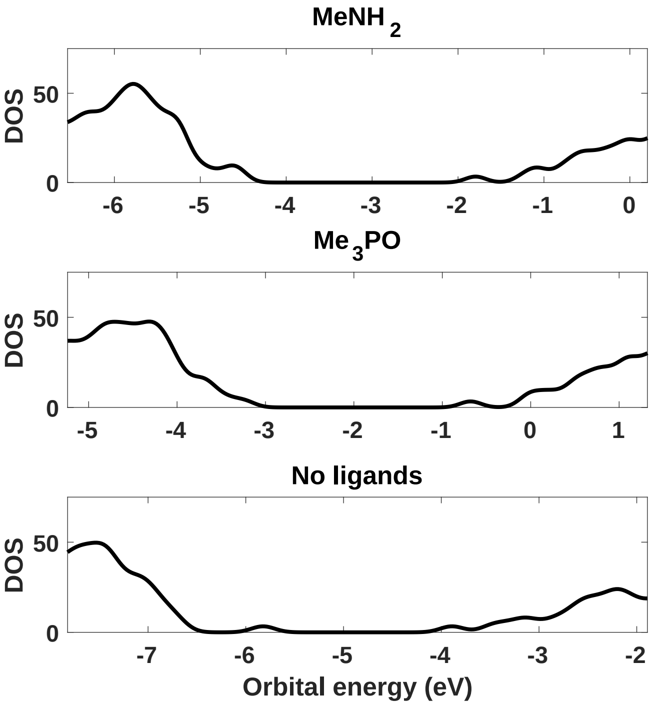

Supplementary Figure 4: Orbital density of states for $Cd_{38}Se_{38}$ with methylamine ligands (top), Me_3PO ligands (middle), and no ligands (bottom).

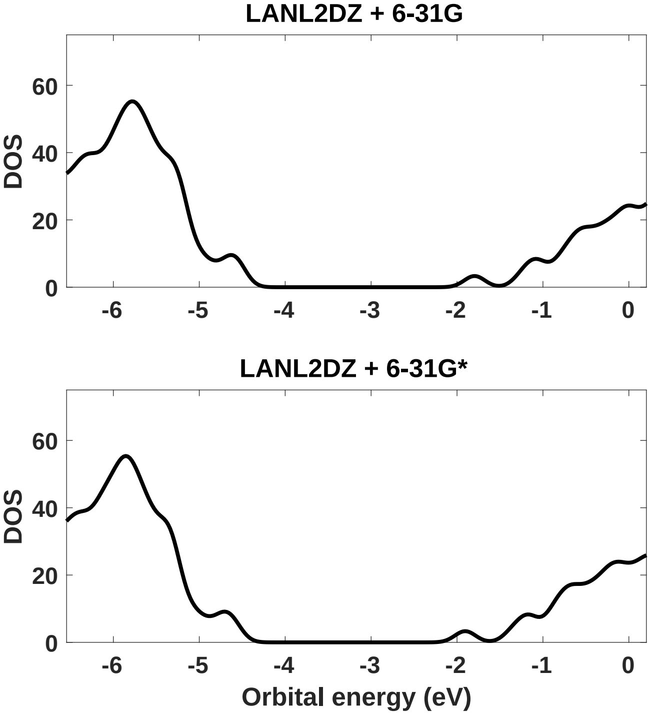

Supplementary Figure 5: Orbital density of states for $Cd_{38}Se_{38}$ with methylamine ligands, using the standard LANL2DZ (top), and LANL2DZ on the Cd and Se with 6-31G* on the ligands (bottom).

a slight shift down in energy upon enlarging the basis set, on average 0.068 eV for the occupied orbitals. The HOMO-LUMO gaps are also in good agreement, with the larger basis set having a gap of just 0.054 eV smaller than the LANL2DZ basis. In Supplementary Figure 6, we show the absorption spectrum and density of transitions with the two basis sets. Due to computational limitations, we were only able to calculate 150 excitations using the larger basis set. The two spectra and densities of transitions are nearly identical, with a small redshift in the 6-31G* results, consistent with the smaller HOMO-LUMO gap.

In Supplementary Figure 7, we show the IPR for the LANL2DZ and 6-31G* results. We find that they again are nearly identical, indicating that the character of the excitations (surface vs bulk) is not changed by the basis set. In Supplementary Figure 8-9, we show the attach and detach densities for representative surface-to-bulk and bulk-to-bulk excitations with the two basis sets. We find that not only is the surface vs bulk character of the excitation unaffected, but visually the excitations look identical across the two basis sets.

We therefore conclude that the results in the LANL2DZ basis are sufficient, and since the 6-31G* basis is significantly more computationally expensive (and too expensive to use on the $Cd_{91}Se_{91}$ NC), we use the LANL2DZ basis for the analysis in the main text.

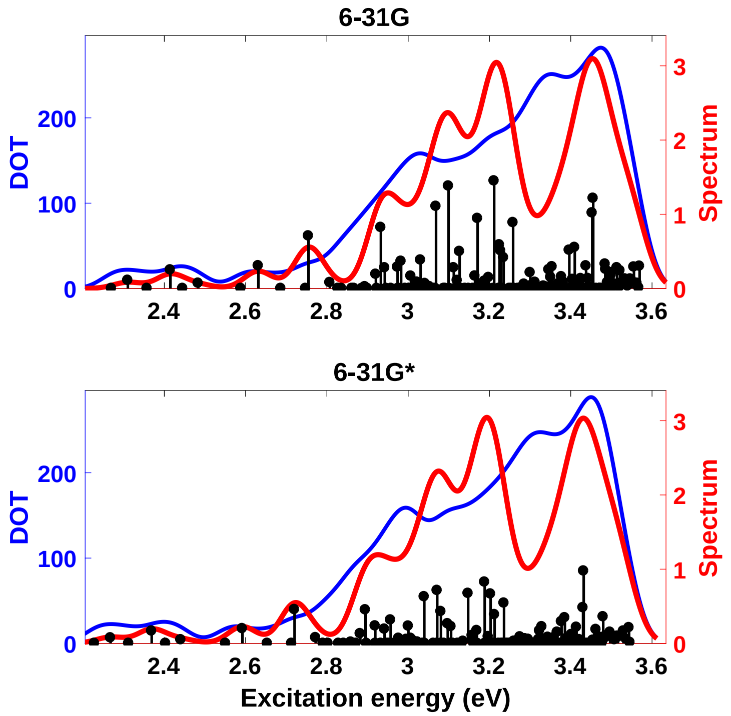

Supplementary Figure 6: (Top) Spectrum (red) and density of transitions (blue) for the first 75 singlet excitations and first 75 triplet excitations of $Cd_{38}Se_{38}$ with methylamine ligands, using the standard LANL2DZ basis set. (Bottom) Spectrum (red) and density of transitions (blue) for the first 75 singlet excitations and first 75 triplet excitations of $Cd_{38}Se_{38}$ with methylamine ligands, using the LANL2DZ ECP for the Cd and Se atoms, but the 6-31G* basis set for the ligands.

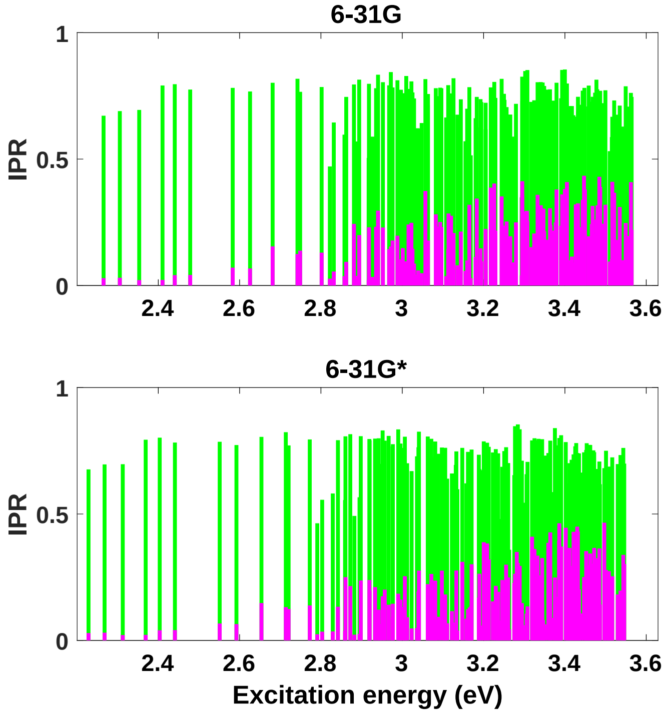

Supplementary Figure 7: (Top) IPR for the electron (green) and hole (magenta) for the first 75 singlet excitations and first 75 triplet excitations of $Cd_{38}Se_{38}$ with methylamine ligands, using the standard LANL2DZ basis set. (Bottom) IPR for the electron (green) and hole (magenta) for for the first 75 singlet excitations and first 75 triplet excitations of $Cd_{38}Se_{38}$ with methylamine ligands, using the LANL2DZ ECP for the Cd and Se atoms, but the 6-31G* basis set for the ligands.

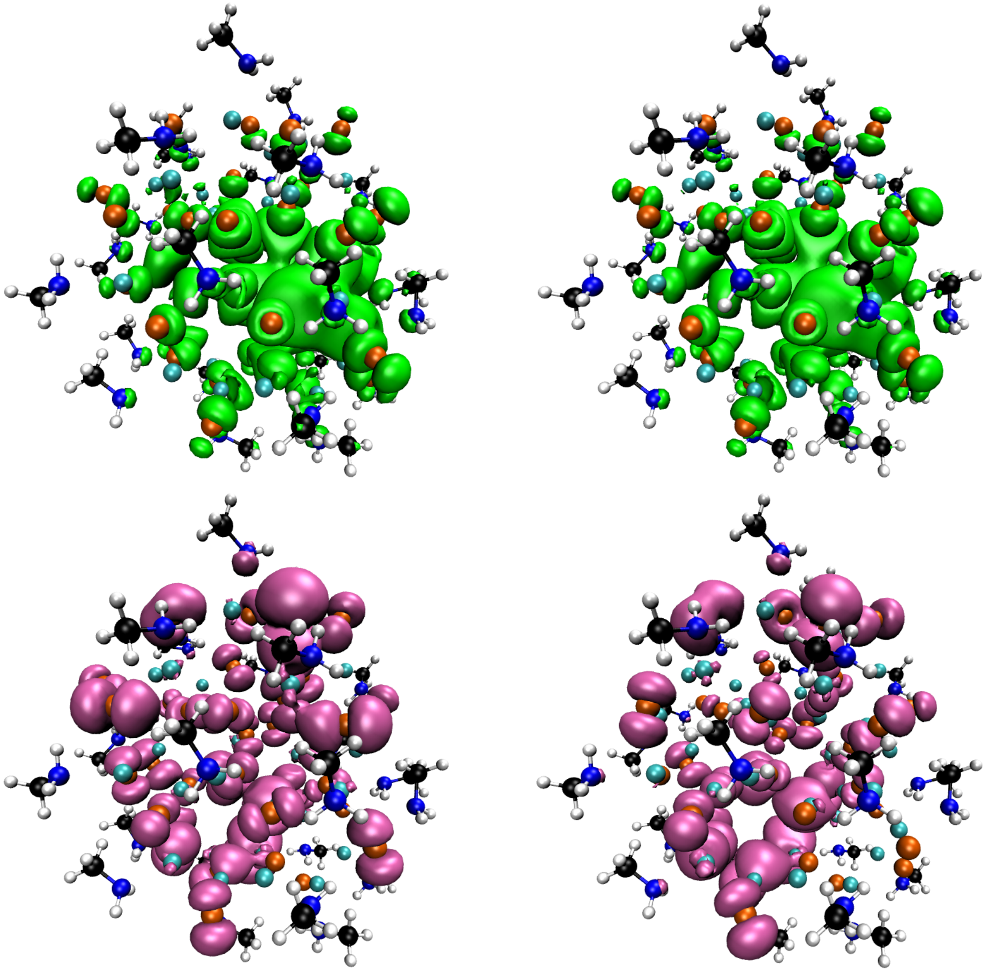

Supplementary Figure 8: Attach (top) and detach (bottom) densities of the first singlet excitation for $Cd_{38}Se_{38}$ with methylamine ligands with standard LANL2DZ (left) and LANL2DZ ECP for the Cd and Se atoms, but the 6-31G* basis set for the ligands (right).

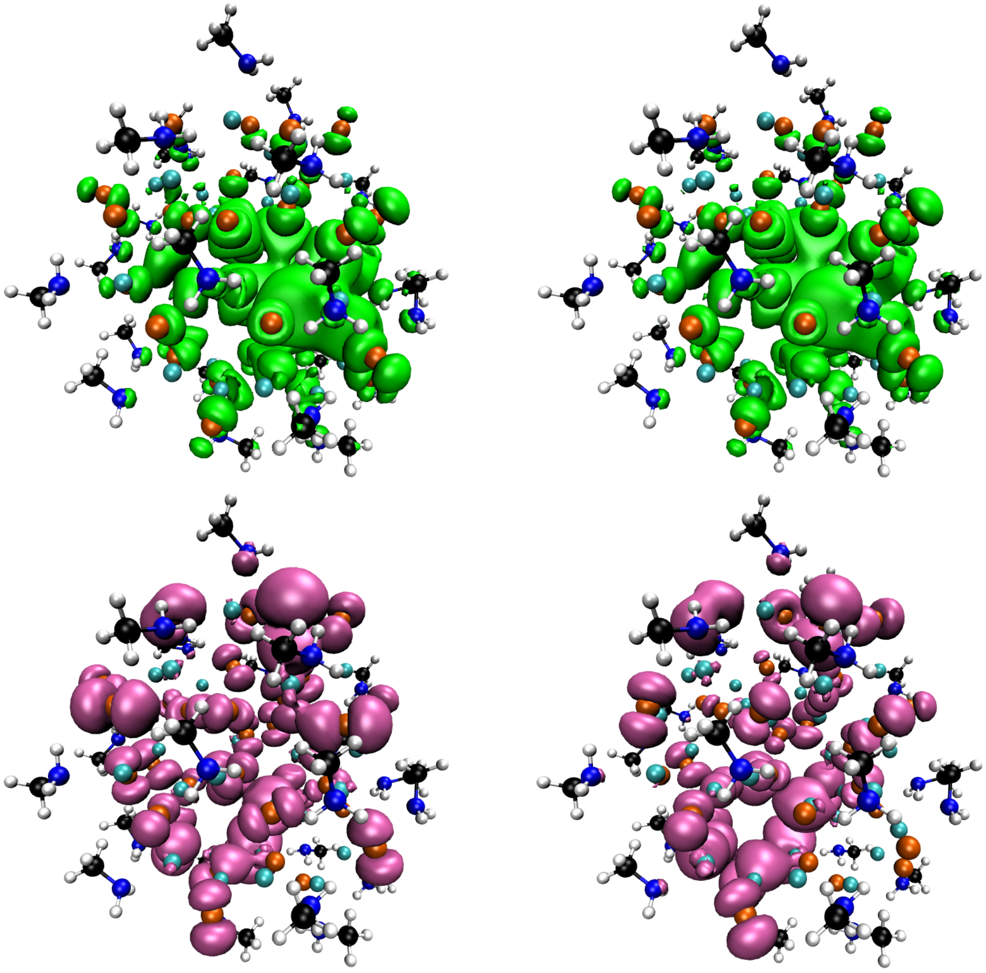

Supplementary Figure 9: Attach (top) and detach (bottom) densities of the brightest excitation in the main peak for $Cd_{38}Se_{38}$ with methylamine ligands with standard LANL2DZ (left) and LANL2DZ ECP for the Cd and Se atoms, but the 6-31G* basis set for the ligands (right).

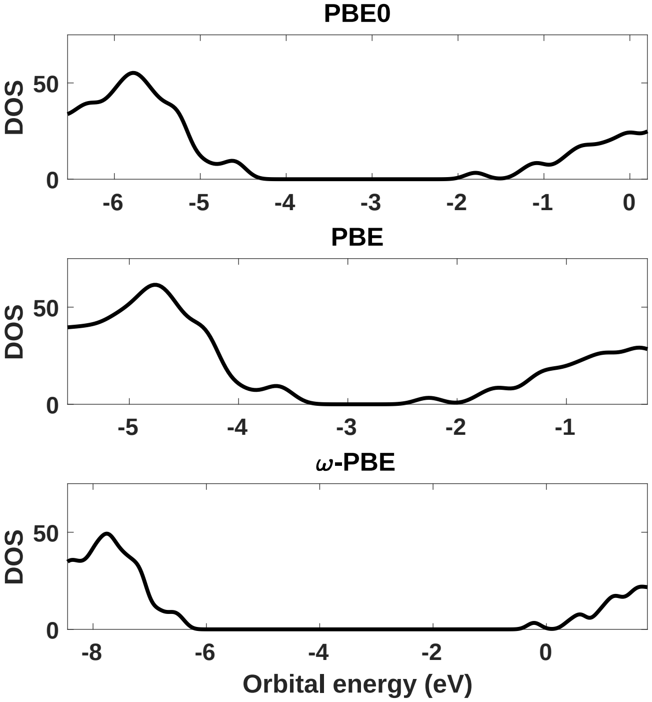

Supplementary Figure 10: Orbital density of states for $Cd_{38}Se_{38}$ with methylamine ligands, using the PBE0 functional (top), PBE functional (center), and ω-PBE functional (bottom).

Supplementary Note 4

Functional comparison We selected the PBE0 functional as it is a hybrid functional which incorporates a fraction of exact exchange, which has been shown to be important for correctly treating the electron-hole interaction in excitonic systems. In order to ensure that our results are not an artifact of the functional chosen, we also conducted TDDFT calculations on the $Cd_{38}Se_{38}$ NC using PBE and ω-PBE. Due to computational limitations, we could only calculate 150 excitations.

In Supplementary Figure 10 we compare the orbital density of states using PBE, PBE0, and ω-PBE. We find that, as expected, PBE has the smallest band gap, PBE0 has a larger band gap, and ω-PBE has the largest band gap. Other than the size of the band gap, qualitatively, all three functionals produce nearly identical orbital density of states.

In Supplementary Figure 11 we compare the density of transitions and absorption spectrum using PBE, PBE0, and ω-PBE. As we would expect from the shifted band gaps, the excitation energies are quite different between the three functionals. We find that all three functionals produce qualitatively similar absorption spectra, with a long tail of dark states, followed by a small side peak, then 3 main bright peaks. PBE and PBE0 show qualitatively similar densities of transitions, while ω-PBE's density of transitions is different due to its asymmetric affect on singlets and triplets.

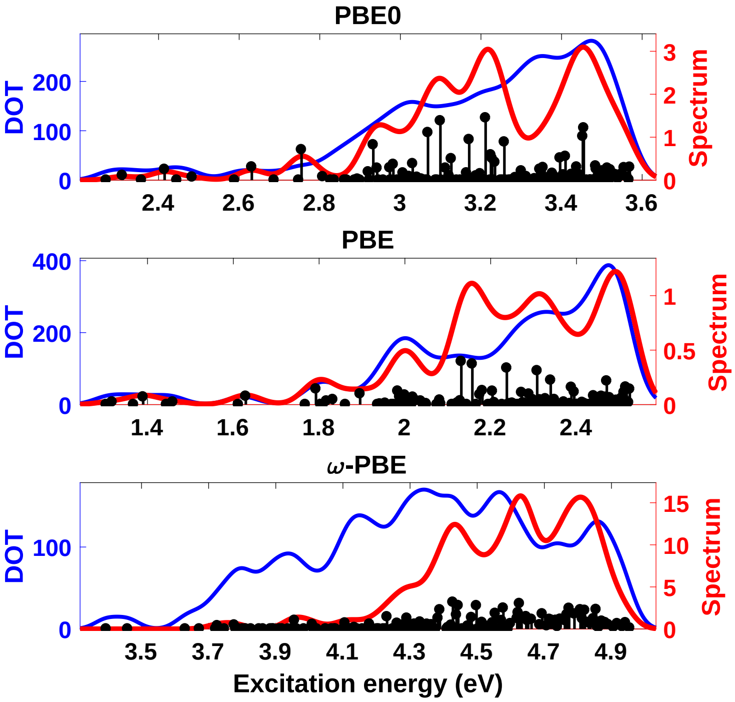

Supplementary Figure 11: Spectrum (red) and density of transitions (blue) for the first 75 singlet excitations and first 75 triplet excitations of $Cd_{38}Se_{38}$ with methylamine ligands, using the PBE0 functional (top), PBE functional (center), and ω-PBE functional (bottom).

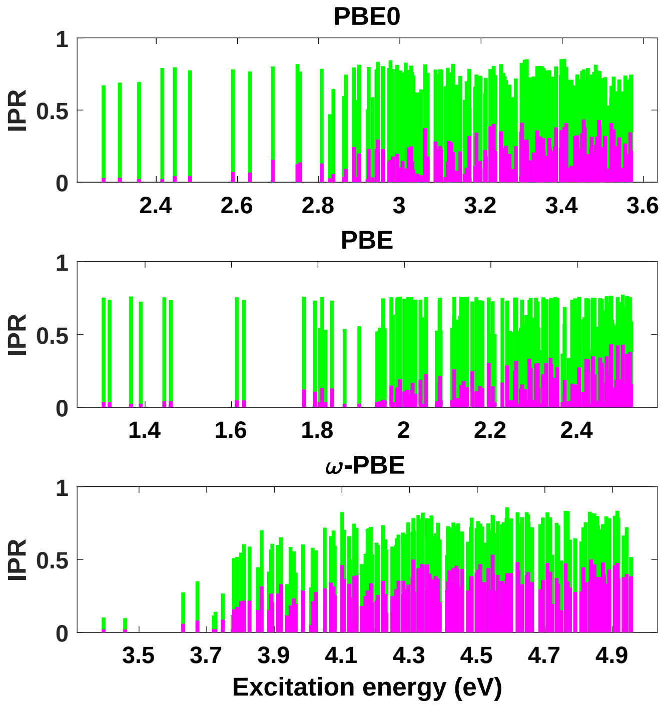

Supplementary Figure 12: IPR for the electron (green) and hole (magenta) for the first 75 singlet excitations and first 75 triplet excitations of $Cd_{38}Se_{38}$ with methylamine ligands, using the PBE0 functional (top), PBE functional (center), and ω-PBE functional (bottom).

In Supplementary Figure 12 we show the charge analysis and IPR for all 3 functionals, and in Supplementary Figure 13-14 we show the attach and detach densities for representative surface-to-bulk and bulk-to-bulk excitations with the three different functionals.

The PBE and PBE0 functionals show very similar results, with low-energy excitations being surface to bulk transitions, and higher-energy excitations being a mix of surface to bulk and bulk to bulk transitions. Comparing the attach and detach densities between PBE and PBE0, we also see that the lower energy PBE excitations are indeed surface to bulk transitions that appear visually identical to the PBE0 excitations. However, in general, the higher energy "delocalized" or "bulk" holes in the PBE calculation appear to be more localized than in the PBE0 calculation, which can also be seen from their lower IPR values.

Comparing PBE0 and ω-PBE, we see from both the IPR and the attach densities that the low-energy electrons are significantly more localized than in the PBE0 calculation, and we see the appearance of surface to surface transitions that were not present in the PBE0 calculation. We also see that the higher energy holes are more delocalized than in PBE0. Despite these differences, the qualitative result remains that the low energy states are localized surface states and the higher energy states are a mixture of surface and bulk excitations.

Because our results are not sensitive to the functional used, we will conduct our simulations in the main text with PBE0, which is more computationally affordable than ω-PBE.

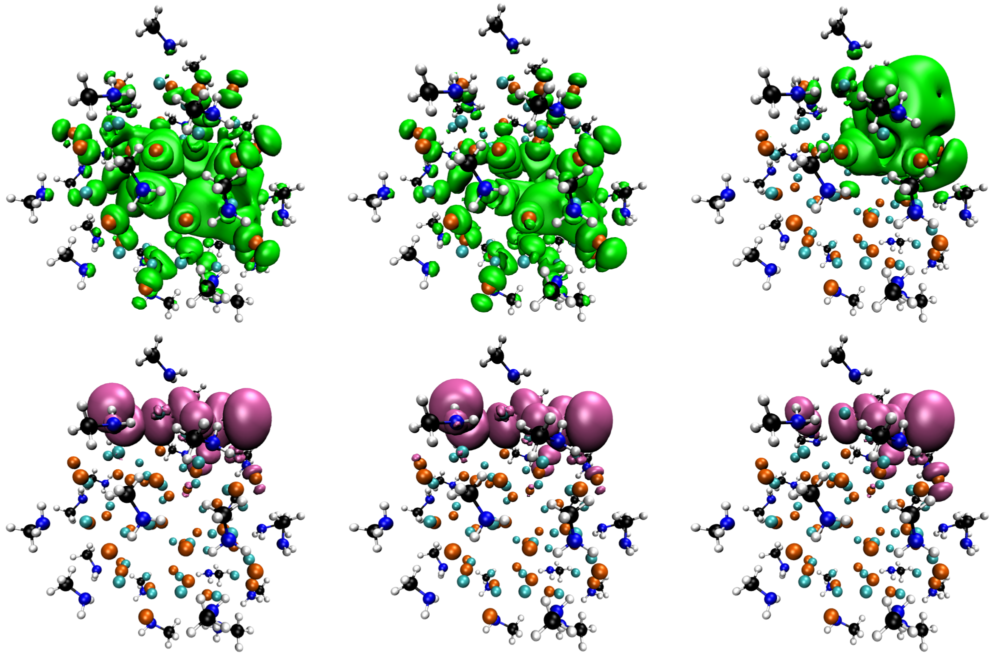

Supplementary Figure 13: Attach (top) and detach (bottom) densities of the first singlet excitation for the $Cd_{38}Se_{38}$ NC with PBE0 (left), PBE (center), and ω-PBE (right)

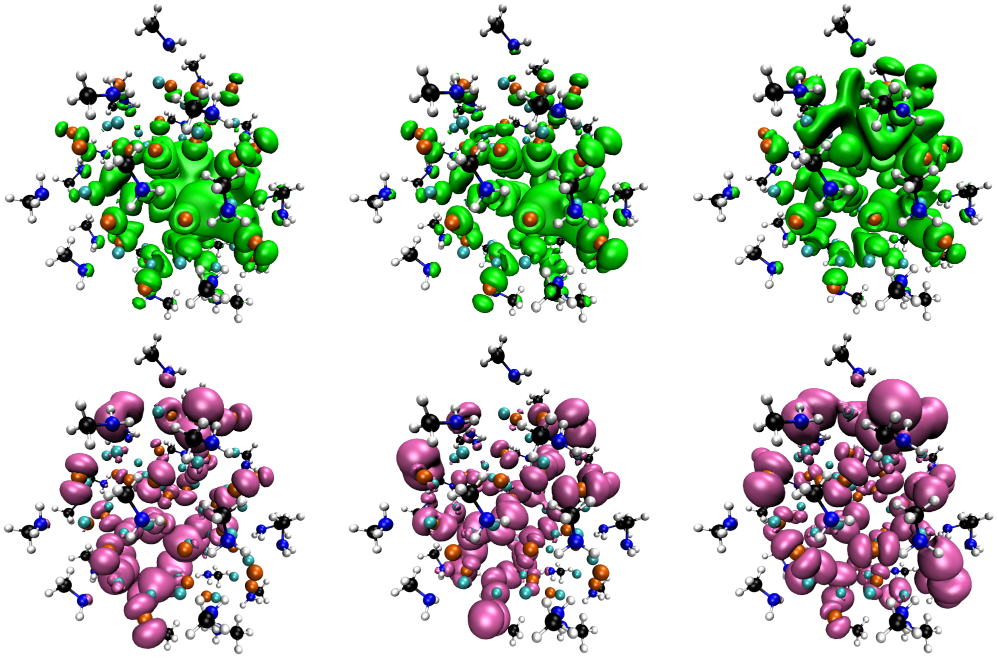

Supplementary Figure 14: Attach (top) and detach (bottom) densities of the brightest excitation from the center peak for the $Cd_{38}Se_{38}$ NC with PBE0 (left), PBE (center), and ω-PBE (right)

Supplementary Note 5

Attach/detach densities with different ligands

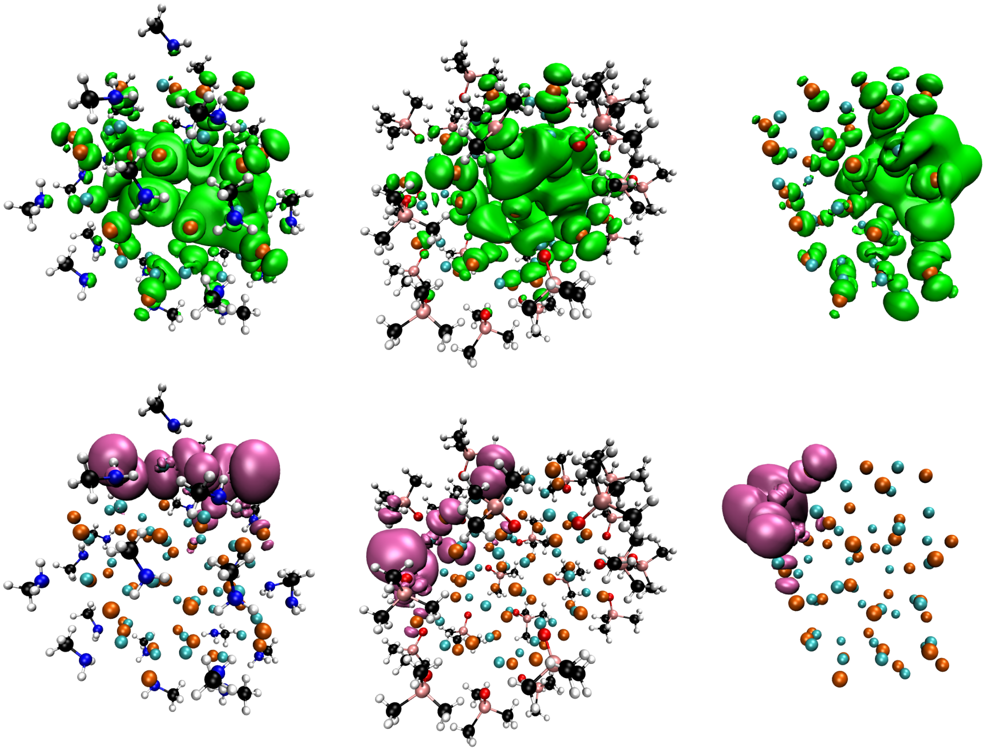

Supplementary Figure 15: Attach (top) and detach (bottom) densities of the first singlet excitation for the $Cd_{38}Se_{38}$ NC with methylamine ligands (left), Me_3PO ligands (center), and no ligands (right).

Supplementary Note 6

Reduced excitation space for $Cd_{91}Se_{91}$ For the TDDFT calculations on $Cd_{91}Se_{91}$, 500 core orbitals were frozen to allow a larger number of excitations to be calculated.

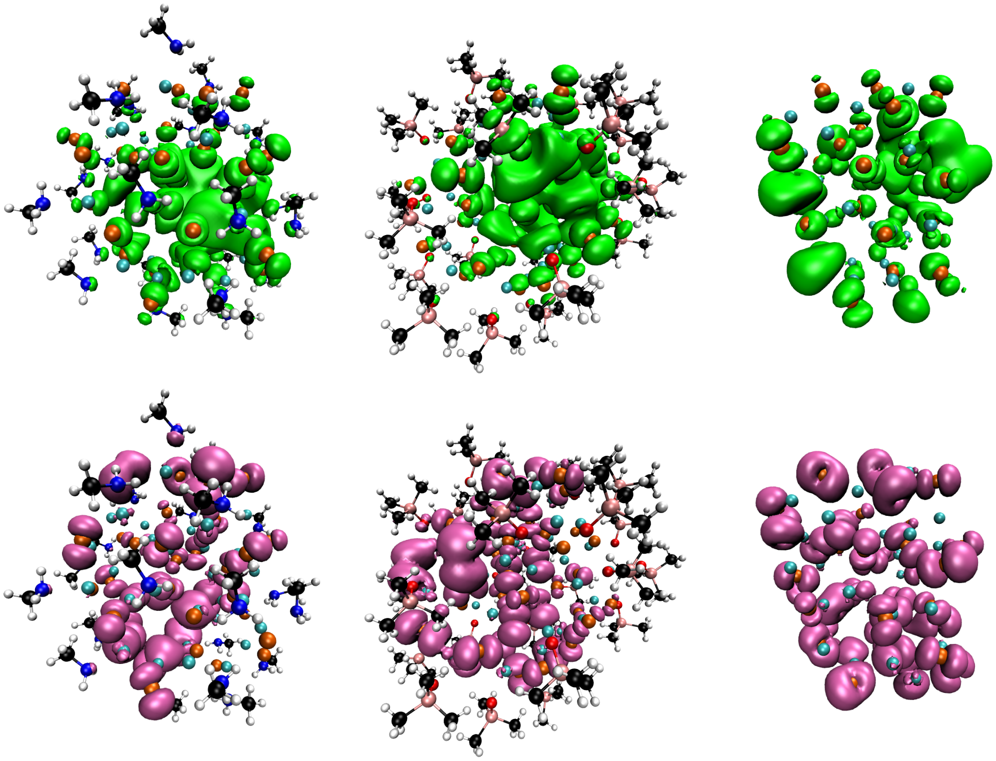

Supplementary Figure 16: Attach (top) and detach (bottom) densities of the brightest overall excitation for $Cd_{38}Se_{38}$ with methylamine ligands (left), Me_3PO ligands (center), and no ligands (right)
