# Supplementary material for: Colloidal CdSe nanocrystals are inherently defective
Source: Nat Commun. 2021 Feb 9;12:890. doi: 10.1038/s41467-021-21153-z (PMC7873310; doi:10.1038/s41467-021-21153-z)
Supplement: Supplementary file 3 — Description of Additional Supplementary Files [file 41467_2021_21153_MOESM3_ESM.pdf]

Description of Additional Supplementary Files

File Name: Supplementary Data 1
Description: Optimized XYZ coordinates for all QD structures.

File Name: Supplementary Data 2
Description: TDDFT and TDA input files for QChem.
